# Orientation of L4 coronal tilt relative to C7 plumb line as a predictor for postoperative coronal imbalance in patients with degenerative lumbar scoliosis

**DOI:** 10.1038/s41598-020-73352-1

**Published:** 2020-09-30

**Authors:** Jiandang Zhang, Zheng Wang, Pengfei Chi, Cheng Chi

**Affiliations:** Department of Spine Surgery, The Chinese PLA General Hospital, 28 Fuxing Rd, Beijing, 100853 China

**Keywords:** Risk factors, Musculoskeletal system

## Abstract

The study design is case–control. To evaluate the impact of preoperative coronal patterns based on the relationship between orientation of L4 coronal tilt and C7 plumb line on immediate postoperative coronal imbalance in degenerative lumbar scoliosis (DLS) patients. Although lumbosacral fractional curve has been long stressed in correction surgery of DLS, there is paucity of literature focusing on preoperative coronal pattern based on the relationship between orientation of L4 coronal tilt and C7 plumb line and its impact on immediate postoperative coronal imbalance in DLS patients. A consecutive series of DLS patients who underwent deformity correction surgery via posterior-only approach were reviewed. According to the relationship between orientation of L4 coronal tilt and C7 plumb line preoperatively, a total of 77 DLS patients who underwent posterior spinal corrective surgery were classified into: 1. Coronal consistency pattern, L4 coronally tilts toward C7 plumb line; 2. Coronal opposition pattern, L4 coronally tilts opposite C7 plumb line. Coronal imbalance was defined as global coronal malalignment (GCM) on either side more than or equal to 20 mm. Whole-spine standing radiographs of both pattern groups were assessed preoperatively and postoperatively. There were 37 patients with coronal consistency pattern and 40 patients with coronal opposition pattern. Compared to patients with coronal opposition pattern, patients with coronal consistency pattern had significantly higher postoperative GCM (*P* = 0.028), lower amount of GCM correction (*P* = 0.013) and higher incidence of postoperative coronal imbalance (*P* = 0.001); further logistic regression analysis revealed coronal consistency pattern was associated with increased odds of postoperative coronal imbalance (odds ratio: 5.981; 95% confidence interval 2.029–17.633; *P* = 0.001). DLS patients with preoperative coronal consistency pattern carried greater risk for immediate postoperative coronal imbalance following posterior long correction surgery.

*Level of evidence* 3

## Introduction

Degenerative lumbar scoliosis (DLS) is a de novo adult scoliosis caused by asymmetric disc degeneration, may present with imbalance in the coronal and sagittal plane^1^. One of the main goals of surgical treatment of degenerative scoliosis is to achieve coronal balance. However, due to the complexity of the structural pathologies of DLS, it might be challenging to achieve satisfactory correction for each DLS patient, resulting in postoperative coronal imbalance. As reported in the literature, the prevalence of postoperative coronal imbalance in adult scoliosis patients ranged from 25.6 to 31.6%^2,3^. In addition, coronal imbalance may also have negative impact on health-related quality of life^2,4^. Therefore, prevention of coronal imbalance after corrective surgery in adult scoliosis patients is of great importance.

Identifying predictive factors for postoperative coronal imbalance is a key step to avoid the occurrence of this complication. However, studies that analyzed risk factors for failed restoration of coronal balance in adult scoliosis patients are few. Bao et al.^2^ showed the preoperative magnitude of coronal imbalance and C7 PL on the convex side of lumbar main curve may influence the surgical outcomes of DLS. Lewis et al.^SPS:refid::bib55^ believed that the ability to level L4 or L5 coronal tilt had great impact on achieving postoperative coronal imbalance after reviewing 46 patients. However, there is paucity of literature focusing on preoperative coronal pattern and its effects on the postoperative coronal imbalance. Due to high incidence of postoperative coronal imbalance, further investigations of coronal imbalance to identify more risk factors are in great needs.

DLS can be viewed as a spinal disorder that involves malalignments at the global, regional, or segmental level in adults^6^. As an integral component of whole spine, lumbosacral fractional curve (L4-S1) at regional spinal level plays an important role in developing coronal imbalance in DLS^7^. In our clinical practice, we found orientation of L4 relative to C7 plumb line (C7 PL) played a role on achieving postoperative coronal balance in DLS patients. Although Lewis et al.^5^ suggested that the magnitude of L4 coronal tilt might be associated with postoperative coronal imbalance, it is still not elucidated how the orientation of L4 coronal tilt relative to C7 PL impacts the postoperative coronal imbalance. In this study, we focused on the orientation of L4 coronal tilt relative to C7 PL to investigate whether the orientation of L4 coronal tilt relative to C7 PL is an independent risk factor for postoperative coronal imbalance.

## Materials and methods

### Patient enrollment

Ethics approval by Chinese PLA General Hospital was obtained before the beginning of this study, and all of the methods were performed in accordance with the guidelines and regulations of the ethics review board. A consecutive series of DLS patients (age > 45 years) who underwent primary spinal deformity correction and long fusion surgeries through posterior-only approach between January 2016 and May 2019 were enrolled. Exclusion criteria included: revision spinal surgeries, fusion levels < 5, history of hip or knee surgery, absolute discrepancy of leg length > 20 mm. Informed consent was obtained from all subjects.

### Operative management

After exposure, bilateral pedicle screws were placed segmentally at every level in the construct. While low instrumented vertebra at L5 was in 25 patients, 52 patients were fused extension to the pelvis, of which, 14 patients were fused to the sacrum with S1 screws only, 7 patients with both S1 screws and iliac wing screws, and 31 patients with both S1 screws and S2 alar-iliac screws. To obtain better deformity correction, spinal osteotomies including Schwab grade I(facetectomy), grade II (Ponte osteotomy) were carried out in every patient. Spinal decompression and transforaminal lumbar interbody fusion (TLIF) were carried out at the caudal portion of lumbar spine (L2-S1) if augmentation of fusion as an assistive anterior column support was necessary, or canal/foraminal/lateral recess stenosis existed.

### Radiographic assessment

Full-spine standing anteroposterior and lateral radiographs were collected preoperatively and at 2 weeks postoperatively or discharge from hospital. The measurements were done by computer software Surgimap (version 2.3.1.1; Spine Software, New York, NY) by two independent spine surgeons and the mean values were collected for analysis. Coronal parameters were as below: (1) Global coronal malalignment (GCM), defined as the horizontal distance between C7 PL and CSVL; (2) L4 coronal tilt, defined as the angle between superior endplate of L4 and the horizontal line^5^; (3) L5 coronal tilt, defined as the angle between superior endplate of L5 and the horizontal line^5^; (4) major Cobb angle, defined as the angle between the superior endplate of the most tilted vertebra cranially and the inferior endplate of the most tilted vertebra caudally. Sagittal measurements were as below: (1) sagittal vertical axis (SVA, the distance between C7 PL and posterosuperior corner of S1); (2) thoracic kyphosis (TK, the angle between superior endplate of T5 and T12); (3) pelvic tilt (PT, a positional parameter); (4) pelvic incidence (PI, an anatomical parameter) and (5) pelvic incidence minus lumbar lordosis (PI-LL).

Since spinal osteotomies were performed in every patient, osteotomy levels and grades were recorded as well. The levels of TLIFs and distribution of upper instrumented vertebra (UIV) were also analyzed.

**Figure 1:**
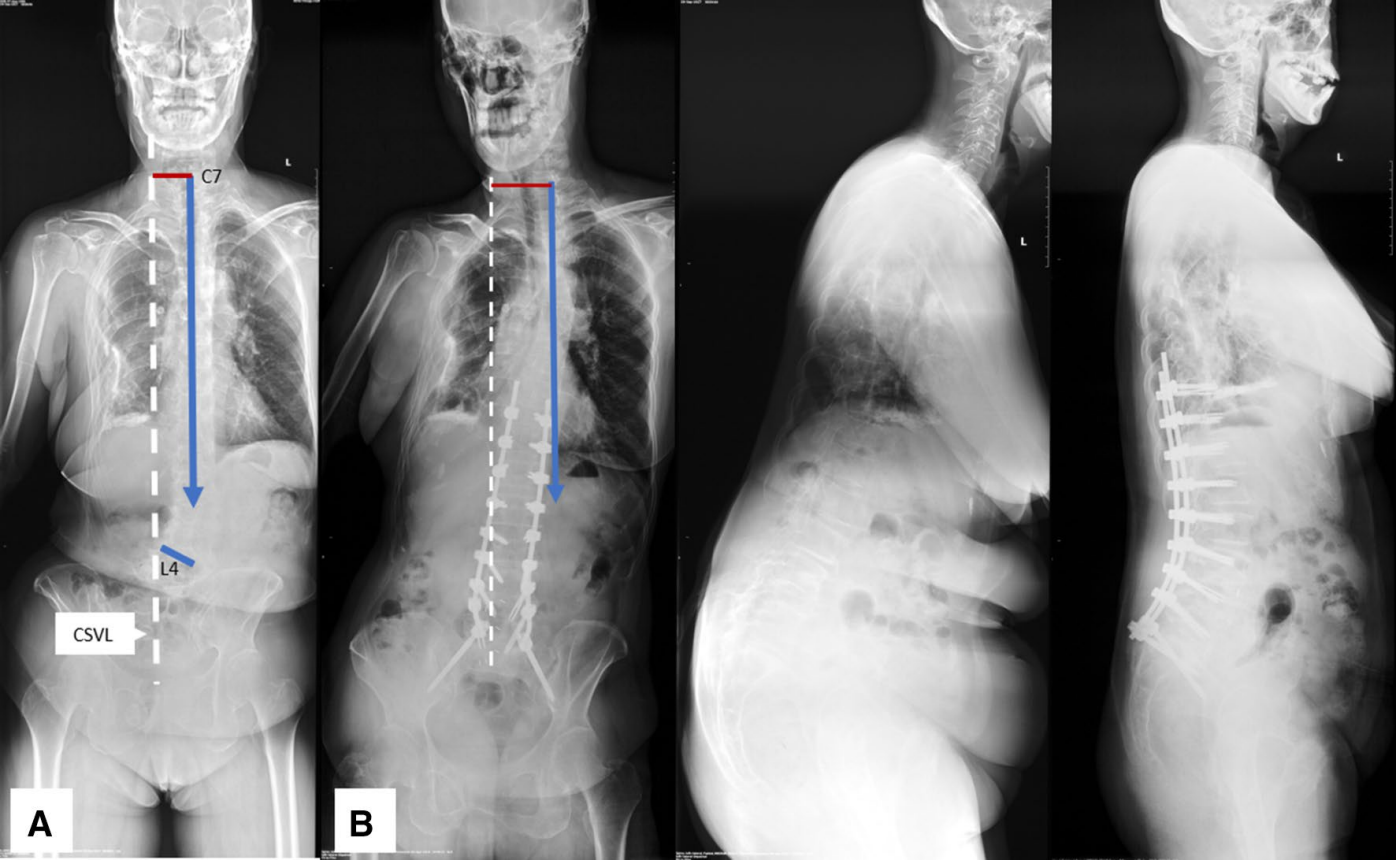
Coronal consistency pattern (**A**). A 67-year-old female with coronal consistency pattern obtained worsening coronal imbalance after surgery (**B**). GCM changed from 44.8 mm preoperatively (**A**) to 58.7 mm postoperatively (**B**), with C7 plumb line on the same side (left) of CSVL. *CSVL* central sacral vertical line, *GCM* global coronal malalignment.

**Figure 2:**
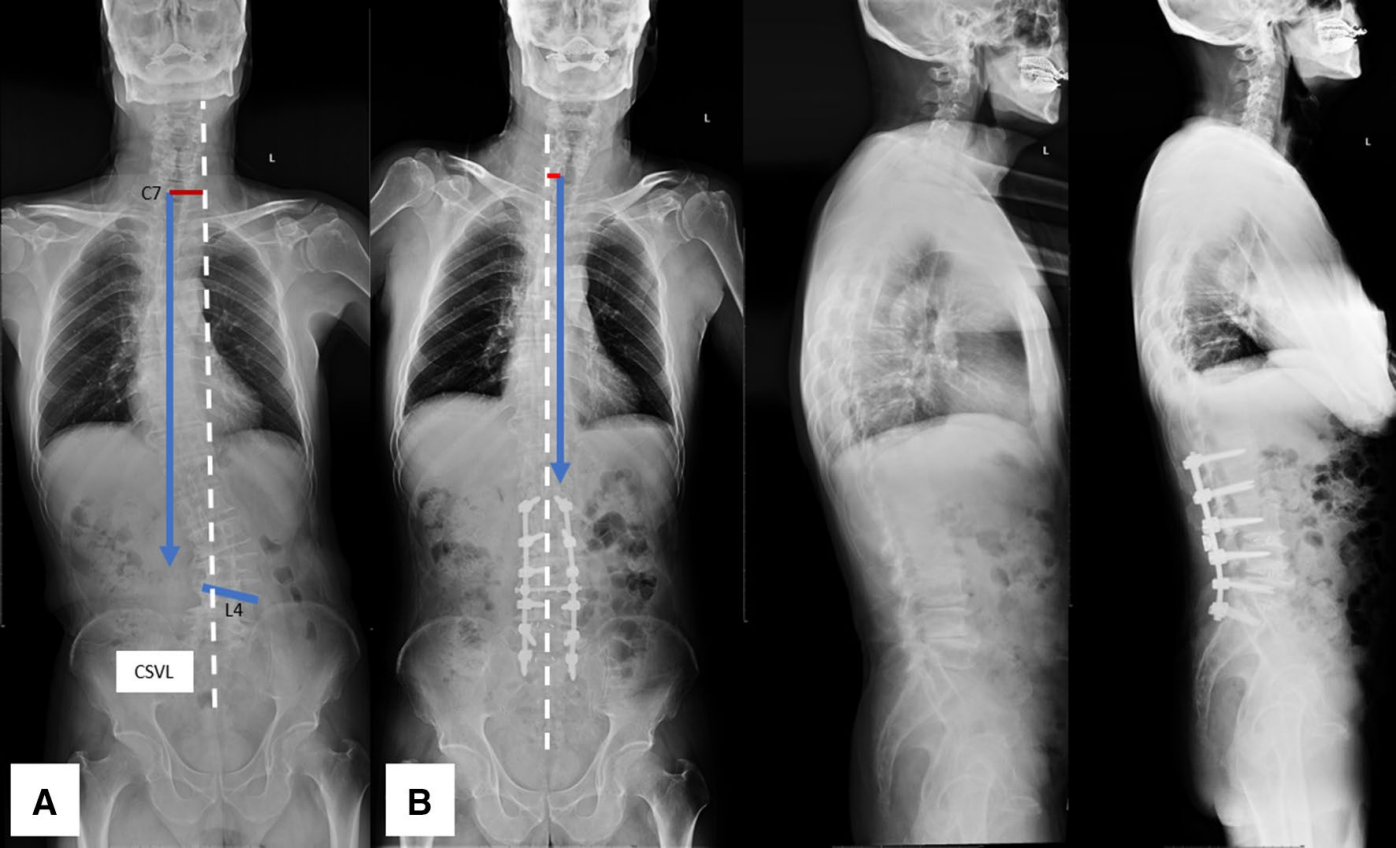
Coronal opposition pattern (**A**). A 71-year-old male with coronal opposition pattern obtained satisfactory correction of coronal imbalance after surgery (**B**). GCM changed from 42.0 mm preoperatively (**A**) to 13.8 mm postoperatively (**B**), with C7 plumb line moved from right side to left side of CSVL. *CSVL* central sacral vertical line, *GCM*: global coronal malalignment.

According to the relationship between orientation of L4 coronal tilt and C7 PL preoperatively, coronal patterns were subdivided into: 1, coronal consistency pattern, L4 coronally tilts toward the C7 PL (Fig. 1A); 2, coronal opposition pattern, L4 coronally tilts opposite the C7 PL (Fig. 2A).

Coronal imbalance was defined as GCM on either side equal to or greater than 20 mm^8^.

### Statistical analysis

Comparison of each variable between the two pattern groups was performed using independent *t* test for continuous variables and Chi-square analysis for categorical variables. Binary logistic regression analysis was further performed to estimate odds ratio for postoperative coronal imbalance^9^. In binary logistic regression analysis, the preoperative coronal opposition pattern was coded as “0”, and consistency pattern as “1”. The statistical analysis was performed using SPSS computer software (version 24; SPSS, Chicago, IL, USA). *P* < 0.05 was set as statistical significance.

## Results

### Comparison of patient characteristics between coronal consistency and opposition pattern groups

There were no differences in age, sex, instrumented levels, UIV, LIV, interbody fusion levels, osteotomy levels and grades between the two pattern groups (Table 1).

**Table 1:** Patient characteristics between two pattern groups.

	Consistency pattern	Opposition pattern	*P* value
Patient No	37	40	
Sex (M:F)	7:30	4:36	0.264^#^
Age at surgery (yr)	64.0 ± 8.0	63.9 ± 6.6	0.953*
Instrumented levels	8.3 ± 2.4	8.7 ± 2.5	0.506*
UIV (T10 or above: below)	23:14	27:13	0.624^#^
LIV (non-pelvic: S1 or below	11:26	14:26	0.622^#^
Interbody fusion levels	2.2 ± 1.2	1.9 ± 1.1	0.259*
Osteotomy grades	1.9 ± 0.3	1.9 ± 0.3	0.909*
Osteotomy levels	3.3 ± 0.8	3.4 ± 0.8	0.776*

*UIV* upper instrumented vertebra, *LIV* lower instrumented vertebra.

*Independent *t* test.

^#^Chi-square test.

### Comparison of preoperative radiological parameters between coronal consistency and opposition pattern groups

The mean value of GCM in patients with coronal consistency pattern was 21.1 ± 17.4 mm, in patients with coronal opposition pattern was 18.1 ± 18.7 mm, but there was no statistically significant difference in preoperative GCM between two groups (*P* = 0.473) (Table 2). There was no significant difference in preoperative major Cobb angle, L4 coronal tilt and L5 coronal tilt. Preoperatively, patients with consistency pattern had 40.5% (15/37) incidence of coronal imbalance, while patients with opposition pattern had 27.5% (11/40) incidence, but there was no statistically significant difference between two groups (*P* = 0.227).

**Table 2:** Comparison of preoperative radiographic parameters between two groups.

	Consistency pattern	Opposition pattern	*P* value
Patient no	37	40	
Preoperative GCM (mm)	21.1 ± 17.4	18.1 ± 18.7	0.473*
Preoperative major Cobb angle (°)	22.9 ± 13.5	25.0 ± 13.7	0.497*
Preoperative L4 coronal tilt (°)	14.0 ± 8.3	11.4 ± 7.2	0.136*
Preoperative L5 coronal tilt (°)	9.2 ± 7.4	7.4 ± 5.3	0.221*
Incidence of coronal imbalance	40.5% (15/37)	27.5% (11/40)	0.227^#^

*GCM* global coronal malalignment.

*Independent *t* test.

^#^Chi-square test.

### Comparison of postoperative coronal alignment parameters and their changes between consistency and opposition pattern groups

Postoperatively, compared to patients with coronal opposition pattern, patients with coronal consistency pattern had significantly higher GCM (20.0 ± 13.1 vs. 14.0 ± 10.0, *P* = 0.028), and less correction in GCM (1.1 ± 20.5 vs. 13.5 ± 22.2, *P* = 0.013) (Table 3). Patients with coronal consistency pattern had significantly higher incidence of postoperative coronal imbalance than those with opposition pattern [ 51.4% (19/37) vs. 15.0% (6/40), χ2 = 11.584, *P* = 0.001]. there was no significant intergroup difference regarding postoperative major Cobb angle, L4 coronal tilt, L5 coronal tilt and their corrections (Table 3). In addition, there was no significant intergroup difference regarding pre- and post-operative sagittal parameters (Table 4).

**Table 3:** Comparison of postoperative radiographic parameters and their changes between two groups.

	Consistency pattern	Opposition pattern	*P* value
Patient no	37	40	
Postoperative GCM (mm)	20.0 ± 13.1	14.0 ± 10.0	0.028*
Postoperative major Cobb angle (°)	8.6 ± 7.3	7.4 ± 5.2	0.392*
Postoperative L4 coronal tilt (°)	5.5 ± 4.9	5.0 ± 4.2	0.582*
Postoperative L5 coronal tilt (°)	4.8 ± 4.2	3.9 ± 3.4	0.274*
Δ GCM (mm)	1.1 ± 20.5	13.5 ± 22.2	0.013*
Δ major Cobb angle (°)	14.5 ± 9.8	17.6 ± 11.7	0.217*
Δ L4 coronal tilt (°)	8.5 ± 7.2	6.2 ± 6.0	0.123*
Δ L5 coronal tilt (°)	4.5 ± 6.7	3.5 ± 4.4	0.470*
Incidence of coronal imbalance	51.4% (19/37)	15% (6/40)	**0.001**^#^

Boldface indicates statistical significance; GCM: global coronal malalignment.

*Independent *t* test.

^#^Chi square test.

**Table 4:** Pre-and post-operative sagittal parameters between two groups (independent *t* test).

	Consistency pattern	Opposition pattern	*P* value
Patient No	37	40	
**Thoracic kyphosis (°)**			
Preoperative	13.8 ± 11.6	14.8 ± 11.3	0.687
Postoperative	21.4 ± 7.9	22.9 ± 9.2	0.451
**Pelvic tilt (°)**			
Preoperative	20.8 ± 11.5	24.9 ± 10.1	0.104
Postoperative	15.9 ± 9.3	16.1 ± 7.7	0.925
Pelvic incidence (°)			
Preoperative	44.9 ± 12.5	45.4 ± 10.9	0.846
Postoperative	45.2 ± 12.2	45.6 ± 11.1	0.891
**PI-LL (°)**			
Preoperative	19.1 ± 19.9	24.0 ± 16.5	0.244
Postoperative	7.2 ± 12.4	4.8 ± 9.6	0.335
**SVA (mm)**			
Preoperative	66.3 ± 48.1	62.1 ± 47.7	0.703
Postoperative	32.5 ± 18.7	28.3 ± 19.2	0.331

*PI-LL* pelvic incidence minus lumbar lordosis, *SVA* sagittal vertical axis.

### Regression analysis

Since Chi-square test results showed that preoperative coronal consistency pattern was a risk factor for postoperative coronal imbalance (χ2 = 11.584, *P* = 0.001), further binary logistic regression analysis was performed to estimate odds ratios for postoperative coronal imbalance, and the results of regression analysis revealed preoperative coronal consistency pattern was associated with significantly increased odds ratio for postoperative coronal imbalance (odds ratio: 5.981; 95% confidence interval 2.029–17.633; *P* = 0.001).

## Discussion

The current study retrospectively evaluated 77 DLS patients who underwent correction surgery via posterior-only approach. Compared to patients with coronal opposition pattern, the results showed patients with coronal consistency pattern had significantly higher postoperative GCM and lower amount of GCM correction, although there was no significant difference regarding preoperative GCM between patients with consistency pattern and those with opposition pattern. Furthermore, patients with coronal consistency pattern had significantly higher incidence of postoperative coronal imbalance than those with opposition pattern (χ2 = 11.584, *P* = 0.001), despite of no significant inter-pattern difference regarding preoperative coronal imbalance. Moreover, further regression analysis revealed coronal consistency pattern was associated with increased odds of postoperative coronal imbalance (odds ratio: 5.981; *P* = 0.001).

It has been reported that preoperative coronal curve types had impact on surgical outcomes in patients with adolescent idiopathic scoliosis^10,11^ and congenital thoracolumbar kyphoscoliosis^12^. Regarding degenerative lumbar scoliosis, Bao et al.^2^ reported that patients with preoperative GCM more than 30 mm and trunk shifting to the convex side of lumbar main curve carried greater risk for postoperative coronal imbalance than those with C7PL shift to the concave side of major curve. The current study demonstrated that preoperative coronal patterns had great impact on postoperative coronal imbalance, that is, the orientation of L4 coronal tilt relative to C7 PL was associated with incidence of postoperative coronal imbalance in DLS patients. Patients with different preoperative coronal consistency or opposition pattern had different surgical outcomes. The incidence of postoperative coronal imbalance in patients with preoperative coronal consistency pattern was significantly higher than those with preoperative coronal opposition pattern (51.4% vs. 15.0%, *P* = 0.001). Further logistic regression revealed preoperative coronal consistency pattern was associated with significantly increased odds ratio for postoperative coronal imbalance (odds ratio: 5.981; 95% confidence interval 2.029–17.633; *P* = 0.001).

Different from mechanisms of postoperative coronal imbalance in degenerative lumbar scoliosis with different preoperative coronal curve types provided by Bao et al.^2^, who believed that asymmetrical osteotomy combined with compression technique on the convex side of the major curve might be the cause, the different surgical outcomes in patients with different preoperative coronal patterns might be due to their different inherent characteristics, regardless of what osteotomy techniques or compression/distraction maneuvers used. In patients with coronal consistency pattern, the orientation of L4 coronal tilt and C7 PL are on the same side of CSVL (Fig. 1A), while the orientation of L4 coronal tilt and C7 PL are on the different side of CSVL in patients with coronal opposition pattern (Fig. 2A). Thus, when a correction surgery is to be performed in patients with coronal consistency pattern, both coronal horizontalization of L4 (lumbosacral fractional curve) and the satisfactory correction of lumbar major curve should be obtained to achieve coronal balance. Otherwise, if either correction of lumbosacral fractional curve or the correction of lumbar major curve is not satisfactory, the coronal imbalance of whole spine might be left in existence (Fig. 1A,B). This might explain why patients with coronal consistency pattern carried greater risk for postoperative coronal imbalance. On the contrary, for patients with coronal opposition pattern, the orientation of L4 coronal tilt and C7 plumb line are on the different side of CSVL. This feature of coronal opposition pattern makes it much easier to correct global coronal malalignment (Fig. 2A,B). This hypothesis was supported by our clinical experience.

In our clinical practice, we found that it was easy to correct the global coronal malalignment in patients with coronal opposition pattern, there were 11 out of 40 cases with whose C7 PL were corrected from one side (right or left) of CSVL preoperatively to the opposite side (left or right) of CSVL postoperatively (Fig. 2A,B). Furthermore, three of which were overcorrected from preoperative coronal imbalance with C7 PL on one side of CSVL to postoperative coronal imbalance with C7 PL on the opposite side of CSVL. During surgery, caution must be taken to avoid overcorrection of global coronal malalignment in patients with coronal opposition pattern. On the contrary, for patients with coronal consistency pattern, it is more difficult to correct the global coronal malalignment. Patients with consistency pattern were more prone to under-correction and left coronal imbalance in existence or even worsening the coronal imbalance on the same side of CSVL as preoperatively (Fig. 1A,B).

The clinical significance of this study lies in that it could help avoid the occurrence of postoperative coronal imbalance. Lumbosacral fractional curve (L4-S1) has long been stressed in spinal deformity correction surgery. Deviren et al.^13^ pointed out that flexibility in the fractional curve had important implications on planning for deformity correction and its evaluation should be a part of surgical decision-making. Brown et al.^14^ found that good postoperative fractional curve correction was correlated with better clinical outcomes in adult scoliosis patients fused to L5 after reviewing 16 patients. Lewis^5^ reported that leveling the L4 coronally was key to achieving coronal balance, with a sample size of 46 patients. Those findings are consistent with our current study, which showed that unsatisfactory horizontalization of L4 in patients with coronal consistency pattern may result in postoperative coronal imbalance. As the most cephalic vertebra in the fractional curve, L4 coronal tilt may represent fractional curve. Coronal horizontalization of L4 reflects the magnitude of correction in fractional curve, it is of great importance during correction of coronal imbalance in patients with coronal consistency pattern, which can provide a leveled foundation to facilitate the correction of the curve(s) above L4. Posterior column osteotomy in the lumbosacral fractional curve plus TLIFs (L5S1, L4/5) could provide effective correction to achieve the coronal horizontalization of L4^15^. On the other hand, in patients with coronal opposition pattern, coronal horizontalization of L4 does not seem as critical as in patients with coronal consistency pattern. Avoiding overcorrection in patients with coronal opposition pattern should be paid attention to.

Limitations of the current study must be mentioned. First, it was a retrospective and small sample-sized study, other confounding factors such as selection bias might affect the results. Second, this study did not involve long-term follow-up or functional scores such as SRS-22 or ODI that reflected the health-related quality of life. A larger sample-sized study with long-term follow-up is needed in the future. Despite these limitations, the current study still demonstrated patients with coronal consistency pattern carried greater risk for postoperative coronal imbalance than those with coronal opposition pattern.

## Conclusion

DLS patients with L4 coronal tilt toward C7 PL were at greater risk for immediate postoperative coronal imbalance than those with L4 coronal tilt opposite C7 PL.

## References

[1] Birknes JK, White AP, Albert TJ, Shaffrey CI, Harrop JS (2008). Adult degenerative scoliosis: a review. Neurosurgery..

[2] Bao H, Yan P, Qiu Y, Liu Z, Zhu F (2016). Coronal imbalance in degenerative lumbar scoliosis: prevalence and influence on surgical decision-making for spinal osteotomy. Bone Joint J..

[3] Ploumis A, Simpson AK, Cha TD, Herzog JP, Wood KB (2015). Coronal spinal balance in adult spine deformity patients with long spinal fusions: a minimum 2- to 5-year follow-up study. J Spinal Disord Tech..

[4] Glassman SD, Berven S, Bridwell K, Horton W, Dimar JR (2005). Correlation of radiographic parameters and clinical symptoms in adult scoliosis. Spine (Phila Pa 1976)..

[5] Lewis SJ, Keshen SG, Kato S, Dear TE, Gazendam AM (2018). Risk factors for postoperative coronal balance in adult spinal deformity surgery. Global Spine J.

[6] Ames CP (2016). Adult spinal deformity: epidemiology, health impact, evaluation and management. Spine Deform..

[7] Campbell PG, Nunley PD (2018). The challenge of the lumbosacral fractional curve in the setting of adult degenerative scoliosis. Neurosurg Clin N Am..

[8] Obeid I, Berjano P, Lamartina C, Chopin D, Boissiere L, Bourghli A (2019). Classification of coronal imbalance in adult scoliosis and spine deformity: a treatment-oriented guideline. Eur Spine J..

[9] Kim TH, Lee SY, Kim YC, Park MS, Kim SW (2013). T1 slope as a predictor of kyphotic alignment change after laminoplasty in patients with cervical myelopathy. Spine (Phila Pa 1976)..

[10] Yang C, Zhao Y, Zhai X, Li J, Zhu X, Li M (2017). Coronal balance in idiopathic scoliosis: a radiological study after posterior fusion of thoracolumbar/lumbar curves (Lenke 5 or 6). Eur Spine J..

[11] Edwards CC, Lenke LG, Peelle M, Slides B, Rihella A, Bridwell KH (2004). Selective thoracic fusion for adolescent idiopathic scoliosis with C modifier lumbar curves: 2- to 16-year radiographic and clinical results. Spine (Phila Pa 1976)..

[12] Xu L (2018). Coronal imbalance after three-column osteotomy in thoracolumbar congenital kyphoscoliosis: incidence and risk factors. Spine (Phila Pa 1976)..

[13] Deviren V, Berven S, Kleinstueck F, Antinnes J, Smith JA, Hu SS (2002). Predictors of flexibility and pain patterns in thoracolumbar and lumbar idiopathic scoliosis. Spine (Phila Pa 1976)..

[14] Brown KM, Ludwig SC, Gelb DE (2004). Radiographic predictors of outcome after long fusion to L5 in adult scoliosis. J Spinal Disord Tech..

[15] Matsumura A (2017). Posterior corrective surgery with a multilevel transforaminal lumbar interbody fusion and a rod rotation maneuver for patients with degenerative lumbar kyphoscoliosis. J Neurosurg Spine..

